# High activation levels maintained in receptor‐binding domain–specific memory B cells in people with severe coronavirus disease 2019

**DOI:** 10.1111/imcb.12607

**Published:** 2022-12-01

**Authors:** Money Gupta, Harikrishnan Balachandran, Raymond H Y Louie, Hui Li, David Agapiou, Elizabeth Keoshkerian, Daniel Christ, William Rawlinson, Michael M Mina, Jeffrey J Post, Bernard Hudson, Nicky Gilroy, Pamela Konecny, Adam W Bartlett, Sarah C Sasson, Golo Ahlenstiel, Dominic Dwyer, Andrew R Lloyd, Marianne Martinello, Fabio Luciani, Rowena A Bull, the COSIN study group

**Affiliations:** ^1^ Faculty of Medicine, School of Medical Sciences University of New South Wales Australia Sydney NSW Australia; ^2^ The Kirby Institute, University of New South Wales, Australia Sydney NSW Australia; ^3^ Antibody Therapeutics Lab Garvan Institute of Medical Research Darlinghurst NSW Australia; ^4^ Serology and Virology Division, Department of Microbiology NSW Health Pathology, Prince of Wales Hospital Sydney NSW Australia; ^5^ Northern Beaches Hospital Frenchs Forest NSW Australia; ^6^ Prince of Wales Clinical School University of New South Wales, Australia Sydney NSW Australia; ^7^ Infectious diseases Royal North Shore Hospital Sydney NSW Australia; ^8^ Infectious Diseases Westmead Hospital Sydney NSW Australia; ^9^ St George and Sutherland Clinical School University of New South Wales, Sydney Sydney NSW Australia; ^10^ Sydney Children's Hospital Randwick Sydney NSW Australia; ^11^ Blacktown Mount Druitt Hospital Blacktown NSW Australia

**Keywords:** SARS‐CoV‐2, RBD, memory B cells, CD11c, CD95

## Abstract

The long‐term health consequences of severe acute respiratory syndrome coronavirus 2 (SARS‐CoV‐2) infection are still being understood. The molecular and phenotypic properties of SARS‐CoV‐2 antigen–specific T cells suggest a dysfunctional profile that persists in convalescence in those who were severely ill. By contrast, the antigen‐specific memory B‐cell (MBC) population has not yet been analyzed to the same degree, but phenotypic analysis suggests differences following recovery from mild or severe coronavirus disease 2019 (COVID‐19). Here, we performed single‐cell molecular analysis of the SARS‐CoV‐2 receptor‐binding domain (RBD)–specific MBC population in three patients after severe COVID‐19 and four patients after mild/moderate COVID‐19. We analyzed the transcriptomic and B‐cell receptor repertoire profiles at ~2 months and ~4 months after symptom onset. Transcriptomic analysis revealed a higher level of tumor necrosis factor‐alpha (TNF‐α) signaling via nuclear factor‐kappa B in the severe group, involving *CD80*, *FOS*, *CD83* and *TNFAIP3* genes that was maintained over time. We demonstrated the presence of two distinct activated MBCs subsets based on expression of *CD80* ^hi^ *TNFAIP3* ^hi^ and *CD11c* ^hi^ *CD95* ^hi^ at the transcriptome level. Both groups revealed an increase in somatic hypermutation over time, indicating progressive evolution of humoral memory. This study revealed distinct molecular signatures of long‐term RBD‐specific MBCs in convalescence, indicating that the longevity of these cells may differ depending on acute COVID‐19 severity.

## Graphical abstract

We performed an in‐depth single‐cell gene expression analysis of the severe acute respiratory syndrome coronavirus 2 (SARS‐CoV‐2) receptor‐binding domain–specific memory B cells in three patients after severe coronavirus disease 2019 (COVID‐19) and four patients after mild/moderate COVID‐19. We analyzed the transcriptome and B‐cell repertoire at ~2 months and ~4 months after symptom onset. The transcriptomic analysis revealed that in the severe group, genes associated with activation, in particular, those associated with tumor necrosis factor‐alpha signaling via nuclear factor‐kappa B were maintained, whereas in the mild/moderate group these declined over time.

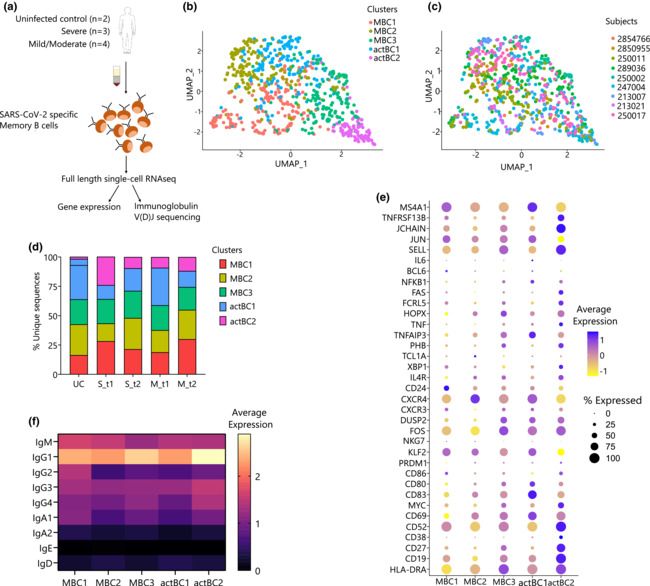

## INTRODUCTION

Since November 2019, the global severe acute respiratory syndrome coronavirus 2 (SARS‐CoV‐2) pandemic has resulted in more than 6 million deaths. Understanding the long‐term immunity and factors associated with protection either through natural infection or vaccination will be key to reducing the long‐term effects of the SARS‐CoV‐2–associated disease, coronavirus disease 2019 (COVID‐19). The early acute phase of severe COVID‐19 has been associated with a delayed and narrow immune response,^1^ as well as a storm of proinflammatory cytokine responses that includes interleukin (IL)‐2, IL‐6, IL‐7, IL‐17 and tumor necrosis factor (TNF)‐α.^2^, ^3^ CD4^+^ T cells skewed toward an inflammatory Th17 phenotype by exposure of IL‐6 and transforming growth factor (TGF)‐β,^4^, ^5^, ^6^ and lymphopenia which has been described as having a varying impact on total CD4^+^, CD8^+^ and B cell composition.^7^ Transcriptomic analysis of CD8^+^ T cells in severe COVID‐19 revealed a highly activated phenotype along with markers of exhaustion and enrichment of pathways linked to costimulation and prosurvival nuclear factor kappa‐light‐chain‐enhancer of activated B cells (NF‐κB) signaling.^8^ Studies have suggested that variations in the immunological memory B cell (MBC) profile between patients with different disease severity ranging from mild to severe are maintained during convalescence.

MBCs are critical for protective immunity because of their ability to rapidly produce neutralizing antibodies upon reinfection and their ability to adapt to specific antigenic profiles.^9^ Encouragingly, several studies have reported the maintenance of T and B cell responses to SARS‐CoV‐2 6–12 months following infection, despite declining neutralizing antibodies in the serum.^10^, ^11^, ^12^, ^13^, ^14^ It is currently unclear how differences in the immune response pertaining to antigen‐specific MBCs (and associated treatments) during acute infection might impact on the longevity and quality of the immunological memory. A study examining MBCs at 1 month after disease onset reported that while all receptor‐binding domain (RBD)–specific B cells had an increased expression of *FcRL5*, the expression of this activation marker was higher following mild disease than following severe disease. The authors speculated that this might associate with increased longevity and a greater likelihood of differentiation into antibody‐secreting cells upon re‐exposure.^12^ However, there are no reports of a detailed molecular analysis of RBD‐specific MBCs in patients with convalescent COVID‐19.

In addition, it is not known whether differences in the maturation of the B cell receptor (BCR) might be impacted by disease severity. The BCR has also been shown to continue to undergo somatic hypermutation (SHM) following resolution of acute COVID‐19, and this maturation is associated with improved affinity for the antigen and neutralization potency; however, it is not known whether this differs with disease severity.^11^, ^15^ Given that severe COVID‐19 has been associated with the loss of germinal centers, CD4^+^ T cells and immunoglobulin M–positive (IgM^+^) B cells, it is possible that BCR maturation may be impaired in those with severe disease in comparison to those with mild disease,^16^, ^17^, ^18^, ^19^ negatively affecting long‐term humoral immunity. Here, we present a detailed analysis of the longitudinal single‐cell RNA transcriptomic and BCR repertoire using peripheral blood mononuclear cells of RBD‐specific MBCs among people who have recovered from either severe or mild/moderate COVID‐19.

## RESULTS

### Isolation of RBD and spike‐specific B cells from patients with COVID‐19

**Figure 1:**
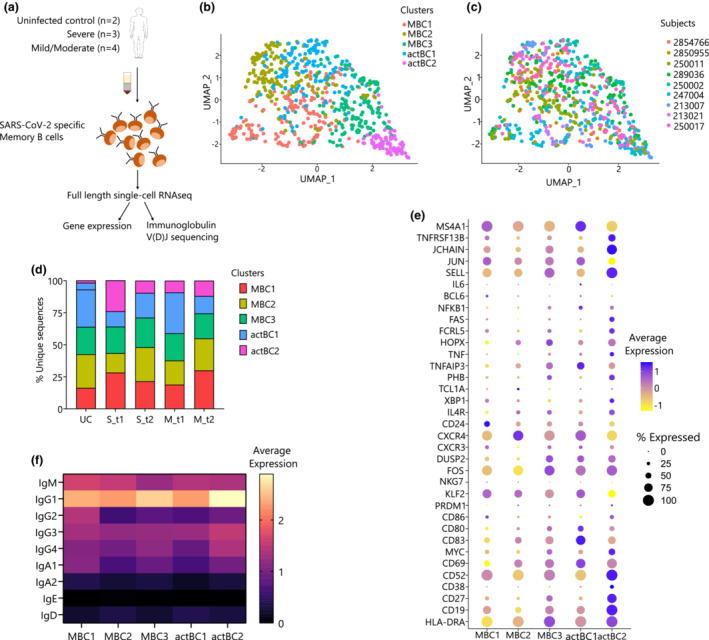
Transcriptomic analysis reveals activated receptor‐binding domain (RBD)–specific memory B cells. **(a)** Experimental design showing the number of patients (*n* = 9) and workflow used in the B cell sorting strategy and single‐cell RNA sequencing (RNA‐seq) pipeline. **(b)** Uniform manifold approximation projection (UMAP) generated using single‐cell RNA sequencing (RNA‐seq) of RBD‐specific memory B cells (MBCs) across five clusters (MBC1, MBC2, MBC2, actBC1 and actBC2). **(c)** UMAP showing distribution of nine patients across two uninfected controls (UCs; 2850955 and 2854766) and seven individuals infected with severe acute respiratory syndrome coronavirus 2 (SARS‐CoV‐2; severe, S group: 289036, 247004 and 250002, mild/moderate, M group: 250011, 213007, 250017 and 213021). **(d)** Stack plot showing distribution of disease severity subtypes in UC, the S group (S_t1 and S_t2) and the M group (M_t1 and M_t2). **(e)** Dot plot showing log‐normalized average expression (color scale) and percentage of expressing cells (size scale) of selected genes across five distinct clusters identified and named as MBC1, MBC2, MBC3, actBC1 and actBC2. **(f)** Average gene expression of isotype transcripts (IgG1, IgG2, IgG3, IgG4, IgA1, IgA2, IgD, IgM and IgE) across the five memory B cell clusters. Ig, immunoglobulin; actBC1, activated MBC cluster 1; actBC2, activated MBC cluster 2; MBC1, MBC cluster 1; MBC2, MBC cluster 2; MBC3, MBC cluster 3.

SARS‐CoV‐2 RBD (*n* = 848) and spike‐specific (*n* = 148) MBCs were single‐cell sorted from blood samples obtained from seven individuals infected with SARS‐CoV‐2 with mild/moderate or severe disease across two time points (Table 1, Supplementary figure 1a, Figure 1a).^10^, ^14^ The first sampling time point (t1) ranged from 49 to 87 days post symptoms (median: 70.5) and the second timepoint (t2) ranged from 110 to 181 days post symptoms (median: 138). The median time between sampling points (t2–t1) was 65 days (range: 58–113 days). Participants had a median age of 52 years (range: 23–84 years), patients with severe disease had a mean age of 60 years and four patients with mild/moderate disease had a mean age of 57.5 years (Table 1). Disease severity was graded based on the National Institutes of Health criteria and participants were allocated to either the severe (S; *n* = 3) group or the mild/moderate (M; *n* = 4) group. Samples from two healthy uninfected controls (UCs) collected prior to the start of the pandemic were used as a comparator group with mean age of 35 years.

**Table 1:** Characteristics of the study population and infection severity

Patient ID	Disease group	Age (years)	Spike^+^ B cells sequenced	RBD^+^ B cells sequenced	Gender	Days post symptom onset		Spike^+^ Bcell frequency (/10^5^ B cells)		RBD^+^ Bcell frequency (/10^5^ B cells)	
						t1	t2	t1	t2	t1	t2
2850955	Uninfected control	26	n/a	n/a	Male						
2584766	Uninfected control	44	n/a	n/a	Female						
250011^a^	Mild	63	105	76	Female	68	181	224	41	47	71
250017^b^	Mild	78		105	Male	73	138	60	85	71	175
213021^f^	Mild	52		51	Male	81	147	28.2	62	7.1	33
213007^g^	Moderate	34	0	146	Male	67	132	202	133	418	44
289036^c^	Severe	84	0	133	Female	87	156	53	67	142	138
247004^d^	Severe	23	0	143	Male	58	116	155	106	329	355
250002^e^	Severe	72	43	121	Male	49	110	40	48	38	64

BD, two times a day; n/a, not available; PRN, pro re nata (as needed); QID, four times a day; RBD, receptor‐binding domain.

^a^ Had obesity and no treatments. SpO_2_ of 98%.

^b^ Had hypertension and was a smoker. SpO_2_ not calculated.

^c^ Telmisartan 80 mg mane; amlodipine 5 mg mane; sitagliptin 100 mg mane; allopurinol 1 tablet every 2 days; Movicol 2 sachets BD PRN for constipation; paracetamol 1 g QID PRN for fever. Had hypertension, diabetes and obesity. Optimal oxygen saturation (SpO_2_) of 71.7%.

^d^ Penrindopril, amlondopril, clotrimazole, prednisone, benzylpenicillin–ceftriaxone–doxycycline, rosuvastatin. Chronic lung disease (asthma). Smoker. SpO_2_ of 92%.

^e^ Ibuprofen prior to admission. Inpatient: paracetamol, nebulized saline. SpO_2_ of 91%.

^f^ No comorbidity or documented treatment. SpO_2_ not calculated.

^g^ SpO_2_ of 99%.

### RBD‐specific memory B cells show transcriptomic heterogeneity

To study the heterogeneity of SARS‐CoV‐2–specific MBCs (*CD19* ^+^ *CD20* ^+^ *CD10* ^−^ *IgD* ^*−*^ *RBD* ^+^) and their distribution across different disease severities, only the RBD^+^IgD^−^ B cells were considered for analysis, as spike^+^ and IgD^+^ cells were not equally sampled across the patients and could skew the single‐cell transcriptomic analysis. This left a total of 732 MBCs after initial quality control, which were composed of 671 RBD^+^ MBCs from the seven infected patients and 61 non‐antigen‐specific MBCs from two UCs that were processed as controls for the single‐cell transcriptomics analysis. Antigen‐experienced MBCs are the type of B cells that have encountered an antigen during initial immune response; however, the B cells isolated for UC were non‐antigen‐specific MBCs (*CD19* ^+^ *CD20* ^+^ *CD10* ^*−*^ *IgD* ^*−*^). After quality control and integration of a scaledTPM matrix, 732 RBD^+^ MBCs across seven patients were corrected for batch before distinct MBC clusters were identified.

Analysis of the gene expression and protein expression (mean fluorescence intensity) data confirmed the presence of class‐switched B cells (IgD^−^) with lower expression of IgD across all clusters (Supplementary figure 1d, Supplementary figure 2). An unbiased single‐cell transcriptomic analysis of RBD‐specific MBCs identified a total of five distinct clusters (Figure 1b). The clusters were distributed heterogeneously across the seven participants infected with SARS‐CoV‐2 (213007, 247004, 250002, 250011, 250017, 213021 and 289036) and two UCs (2850955 and 2854766) (Figure 1c). All clusters expressed a similar number of genes with an average median expression of 2155 genes (range: 1748–2903).

Differential gene expression (DGE) analysis reported 591 genes that were differentially expressed across five distinct clusters with adjusted *P*‐value < 0.05 (Supplementary table 1). The first cluster was enriched for heavy‐ and light‐chain genes, such as *IGLC2*, *IGHG2*, *IGLC3* along with *PLAC8*. Gene set enrichment analysis (GSEA) showed no pathways that were enriched for this cluster, so this cluster was named as MBC cluster 1 (MBC1; Supplementary table 2). The MBC1 cluster decreased proportionally over time in the S group from 28.30% to 21.47%, whereas it increased in the M group from 18.86% at t1 to 30% at t2 (Figure 1d, Supplementary figure 1b).

The second cluster termed as MBC cluster 2 (MBC2) showed significantly higher expression of *CXCR4*, *TXNIP*, *MT‐ND2* and *RPS27*, but did not have enrichment of any pathways with *P*‐values < 0.05. MBC2 increased over time in both the disease groups: from 15.09% to 26.55% in the S group and from 18.86% to 25% in the M group.

The third cluster termed as, MBC cluster 3 (MBC3), presented DGE of *SELL*, *LTB*, *IGKC*, *S100A10*, *PSME2* and *CTSH*. This cluster was found to be enriched in pathways associated with fatty acid metabolism with leading edge genes, such as *S100A10*, *LDHA*, *OSTC*, *UROD* and *UROS* (Supplementary table 2). Similar to MBC1, this cluster increased in the S group overtime but decrease in the M group from 21.14% at t1 to 19.38% at t2.

The fourth cluster was significantly enriched for genes associated with immune activation and proliferation, including *TNFAIP3, CD83, JUNB, NFKBID* and *NFKBIA*, and hence this cluster was designated as activated MBC cluster 1 (actBC1; Figure 1e, Supplementary figure 1b). The GSEA results indicated actBC1 was associated with the activation and costimulation pathways involved in TNF‐α signaling via the NF‐κB pathway (Supplementary table 2) which has been associated with the longevity of immune responses.^20^, ^21^ Leading edge genes were *DUSP2*, *CD83*, *NFKBIE*, *NFKBIA*, *CD80*, *CD69*, *KLF6*, *NFKB1* and *ZFP36*. It is worth noting that despite an increase in activation markers, including *CD83* and *TNFAIP3*, this cluster had a low expression of *ITGAX*, which encodes for CD11c (Figure 1e, Supplementary figure 2). This cluster had a higher expression of the same genes (*CD83, FOS, DUSP2, MYC* and *CD69*) as a subset previously described in malaria as proliferating MBCs^22^ (Figure 1e). Analysis of the proportion of this cluster across the different disease groups indicated that the actBC1 cluster was present at a lower amount at t1 in the S group when compared with both the UC and M groups (Figure 1d).

Interestingly, the fifth cluster showed the highest number of differentially expressed genes, which comprised 93.06% of the total differentially expressed genes across clusters. This cluster, termed as activated MBC cluster 2 (actBC2), was enriched in several genes representing an activation phenotype comprising *ITGAX* (CD11c) and *FAS* (CD95) that has been previously associated with activated MBCs in COVID‐19^23^, ^24^ and influenza,^25^ along with age‐associated B cells^24^ (Supplementary table 1).

Other genes associated with this cluster were *PIM3*, *CYC1*, *DECR1* and *PRDX3* and indicated enrichment of the adipogenesis pathway^26^ (Supplementary figure 1b). *FcRL5* gene expression was also found to be higher in this group compared with other clusters (Wilcoxon rank sum test: MBC1:actBC2, *P* = 0.0292; MBC2:actBC2, *P* = 0.0002; MBC3:actBC2, *P* = 0.0666; actBC1:actBC2, *P* = 0.0027; Supplementary figure 2).

The actBC2 cluster was mostly composed of cells from patients with severe (33.50%) SARS‐CoV‐2, and only 21.02% in the M group. Compared with actBC1, this cluster had an increased expression of *FAS* and *ITGAX*, separating this activated cluster from the actBC1 MCB population (Supplementary figure 2).

As aging is associated with altered B‐cell phenotypes and the presence of age/autoimmune‐associated B cells defined by the expression of T‐bet^+^ and CD11c^+^, we compared the composition of the clusters between the ages of < 40 (*n* = 3) and > 40 years (*n* = 6). Age/autoimmune‐associated B cells–like cells have been previously described with a higher expression of *ITGAX* (CD11c) in mice and humans,^24^, ^27^ but in this study aging was not associated with an increase in the actBC2 population, and therefore was not a confounder for the increased representation in the S group (Supplementary figure 1c).

### RBD‐specific MBCs in patients with severe COVID‐19 maintain an activated phenotype over a period of 4 months

**Figure 2:**
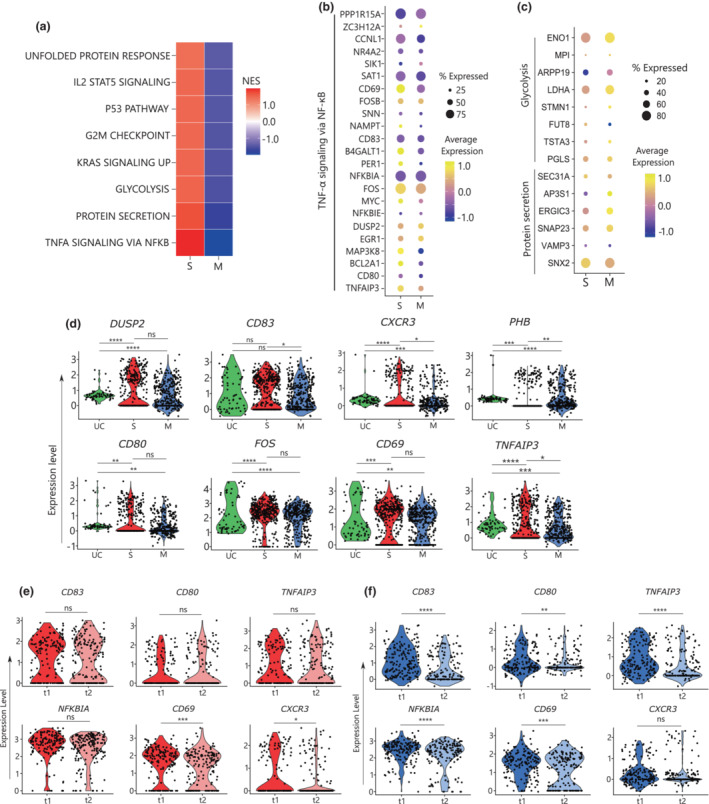
Higher and maintained gene expression profile associated with B cell activation. **(a)** Gene Set Enrichment Analysis (GSEA) showing normalized enrichment score (NES) across the severe (S; *n* = 3) and mild/moderate (M; *n* = 4) groups with NES shown as gradient from blue to red. **(b)** Dot plot showing log‐normalized average expression of genes in tumor necrosis factor‐alpha (TNF‐α) signaling via the nuclear factor‐kappa B (NF‐κB) pathway and the genes involved in **(c)** protein secretion and glycolysis. **(d)** Violin plots of the distribution of activated genes (*CD69*, *CD80*, *CD83*, *CXCR3*, *DUSP2*, *FOS*, *PHB*, *TNFAIP3*) across uninfected controls (UCs; *n* = 2), the severe (S) group (*n* = 3) and the mild/moderate (M) group (*n* = 4). **(e)** Violin plots showing change in the expression level over time of *CD83*, *CD80*, *TNFAIP3*, *NFKBIA*, *CD69* and *CXCR3* in the S and **(f)** M groups. Statistical differences across disease severity and timepoints were calculated using the unpaired two‐tailed Wilcoxon rank sum test with *P*‐values as *P < 0.05, **P < 0.01, ***P < 0.001 and ****P < 0.0001. ns, nonsignificant.

We further looked at the DGE analysis between the S and M groups, t1 and t2 combined, which revealed 1169 differentially expressed genes (Supplementary table 3). Many of these genes were associated with immune activation and proliferation, such as *CD83, FOS, AHNAK* and *MAP3K8* and costimulation genes such as *TNFAIP3* and *NFKBIA*. The GSEA revealed enrichment of several pathways in the S group that were involved in protein secretion and TNF‐α signaling via NF‐κB (Figure 2a, Supplementary table 4). Increase in the TNF‐α signaling via the NF‐κB pathway has been previously shown to be increased in individuals with severe disease and higher levels of proinflammatory cytokines.^2^ The genes involved in this pathway that were highly expressed in the S group in comparison with the M group were *TNFAIP3*, *CD80*, *CD83*, *MYC*, *DUSP2*, *FOS* and *CD69* (Figure 2b). Other pathways that were enriched in the S group were protein secretion and glycolysis. The genes included in the protein secretion pathway were *SNX2*, *VAMP3*, *SNAP23*, *ERGIC3*, *AP3S1* and *SEC31A*. The genes included in the glycolysis pathway were *PGLS, TSTA3, FUT8* and *STMN1* (Figure 2c).

To determine whether the higher activation status of MBCs in the S group is maintained or lost over time, the DEG analysis was performed between t1 and t2 in the S and M groups separately, which revealed 157 and 284 differentially expressed genes, respectively (Supplementary table 9 and Supplementary table 11). In the S group it was noted that many of the activation markers were maintained (Figure 2e), except for *CD69* and *CXCR3*, which was in contrast to the M group, where a decrease in expression from t1 to t2 was observed (Figure 2f). The GSEA of these DEGs identified a loss of TNF‐α signaling via the NF‐κB and interferon gamma response pathways from t1 to t2 in the M group (Supplementary table 10, Supplementary figure 1f) but no significant change in TNF‐α signaling via NF‐κB and inflammatory response was observed in the S group (Supplementary table 12).

In addition, we compared DEGs across the S and M groups at t1 and t2 separately, and identified 141 and 27 DEGs, respectively, with adjusted *P*‐value < 0.05 (Supplementary table 13 and Supplementary table 15). The GSEA of t1 showed enrichment of the interferon‐alpha and interferon‐gamma pathways in the M group compared with the S group (Supplementary table 14). By contrast, at t2 the S group was enriched for the TNF‐α signaling via the NF‐κB and xenobiotic metabolic pathways in comparison to the M group at *P*‐value < 0.05 and normalized enrichment score > 0 (Supplementary table 16, Supplementary figure 3). In summary, this result revealed an increased activation status of MBCs in the S group, and this status was maintained from t1 to t2.

To understand how these profiles compare with other non–SARS‐CoV‐2 MBCs, we examined separately the transcriptomic differences in MBCs between the UC group and the two COVID‐19 groups. A total of 1335 DEGs between the UC and S groups, and 1602 between the UC and M groups, were identified (Supplementary table 5 and Supplementary table 6). Many of these DEGs were part of the activation and proliferation pathways; for example, in the S to UC group comparison, *CD80* and *CXCR3* were increased, whereas *TNFAIP3* and *PHB* were decreased. For the M to UC group comparison, *DUSP2*, *CD83*, *CXCR3*, *CD80*, *FOS, TNFAIP3* and *CD69* were decreased (Figure 2d). GSEA between the UC and S groups showed enrichment of metabolic pathways such as xenobiotic, fatty acid metabolism, adipogenesis and UV response in the S group that are downregulated in UC (Supplementary table 7). Further, comparison of the UC and M groups showed similar metabolic pathways such as glycolysis, oxidative phosphorylation, DNA repair and adipogenesis that were enriched in the M group (Supplementary table 8).

### Gene usage in RBD‐specific memory B cells

In addition to the transcriptomic analysis, successful reconstruction of paired heavy‐ and light‐chain gene BCR sequences was achieved with 95.88% of the spike and RBD MBCs (Supplementary table 17). For this analysis additional 276 IgD^+^ B cells were also included that were excluded from the transcriptomic analysis. An evolutionary distribution of antigen‐specific BCRs showed a diverse repertoire across the two epitopes (spike and RBD), all seven patients and both timepoints (t1 and t2) (Supplementary figure 4a). Most cells were IgG1 (t1 = 55.16%, t2 = 50.99%), IgA1 (t1 = 5.89%, t2 = 4.08%) and IgM (t1 = 12.48%, t2 = 5.72%; Supplementary figure 4b). From all seven patients a subset of IgD^+^ cells was also sequenced from t1 (156/751) and t2 (34/751); however, this subset was not equally represented across patients (Table 1).

**Figure 3:**
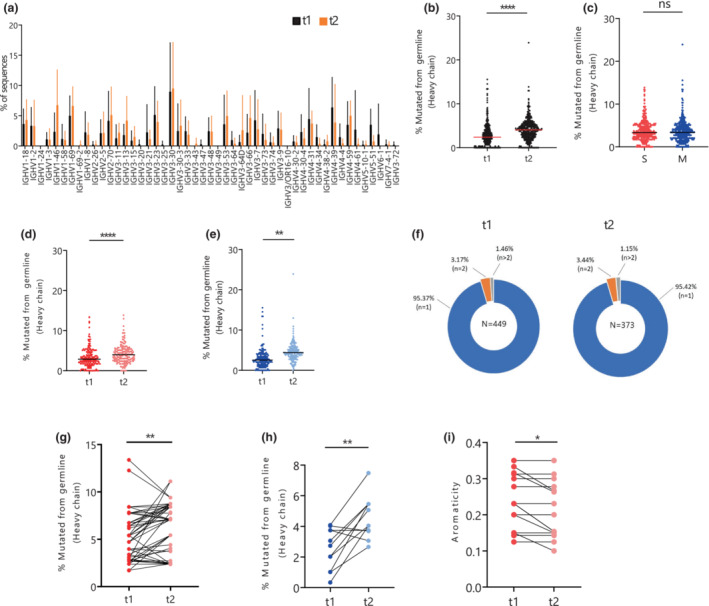
Maturation of receptor‐binding domain (RBD)–specific memory B cells over time. **(a)** Frequency of gene usage, as a percentage, of heavy chains across seven patients at t1 and t2. **(b)** Percentage mutated from germline in IgD^−^ heavy chain across t1 and t2 in both severe (S; *n* = 3: 289036, 247004, 250002) and moderate (M; *n* = 4: 250011, 213007, 250017 and 213021) groups. **(c)** Percentage mutated from germline in IgD^−^ heavy chain in the S and M group in both time points combined and **(d)** between t1 and t2 in the S and **(e)** M groups. **(f)** Percentage of clonal B cell receptor (BCR) population (singlets, *n* = 1; double, *n* = 2; more than two BCRs in a clone, *n* > 2) at t1 and t2 across seven individuals infected with severe acute respiratory syndrome coronavirus 2 (SARS‐CoV‐2). **(g)** Increase in the percentage mutated from germline in heavy chain over time clones of the S and **(h)** M groups of individuals infected with SARS‐CoV‐2. **(i)** Decrease in aromaticity of CDR3H sequences from t1 to t2 over time clones of the S group. Statistical differences across disease severity were calculated using the two‐tailed unpaired *t*‐test (Mann–Whitney *U*‐test), with adjusted *P*‐values as *P < 0.05, **P < 0.01, ****P < 0.0001, ns, nonsignificant. The red horizontal line depicts median at that stage of disease. Nonparametric paired *t*‐test (Wilcoxon) was performed for statistical significance analysis for testing change in percentage mutation from germline and aromaticity in paired over time clones. Ig, immunoglobulin.

Of the 968 BCRs reconstructed, 866 unique clones were identified, reflecting a polyclonal MBC response toward SARS‐CoV‐2 (Supplementary table 18). The gene usage in t1 and t2 was comparable with both timepoints showing dominant use of Vh3‐30 (Figure 3a) as previously reported.^28^ We observed a trend of an increase in HV1 family and a decrease in HV4 family gene usage over time (Supplementary figure 4e) across t1 and t2 but this was not significant (HV1: *P* = 0.2593, HV4: *P* = 0.0973).

For the light chain, 786 sequences were obtained from 996 cells comprising 456 kappa chain and 330 lambda chain. Our single‐cell data showed a high percentage of MBCs with both productive kappa and lambda transcripts (81/996 = 8.13%) within a single cell. However, in previous studies, about 0.5–2% of MBCs have been reported with the presence of both kappa and lambda chains.^29^, ^30^ Interestingly, in six of the seven patients the dual kappa–lambda transcripts decreased over time by approximately half (Supplementary table 17).

KV1‐39, KV3‐20 and LV3‐21 were dominant across both timepoints (Supplementary figure 4c, d). KV1 usage increased and KV3 usage decreased, while other gene family usage remained the same with no statistical difference between t1 and t2 (Supplementary figure 4f; KV1: *P* = 0.0728, KV3: *P* = 0.208). In the case of the light chain, there was a nonsignificant trend over time where LV1 and LV7 usage increased and decreased over time, respectively (Supplementary figure 4g) (LV1: *P* = 0.1563, LV7: *P* = 0.250), whereas LV2 and LV3 showed a significant increase and decrease, respectively, in their gene usage overtime (LV2: *P* = 0.0313, LV3: *P* = 0.0156).

### Maturation of IgD^−^ RBD‐specific memory B cells

Ongoing B cell maturation has been reported in the convalescent phase of SARS‐CoV‐2 infection.^11^ Therefore, we analyzed the mean SHM in switched IgD^−^ MBCs over time and revealed a significant increase in mutation from germline in heavy chain across all the individuals from 2.389% to 4.05% (*P* < 0.0001; Figure 3b). Similar results were observed in the kappa and light chains that presented a significant increase in SHM from 1.65% to 2.465% (*P* < 0.0001) and 1.701% to 2.541% (*P* < 0.0001), respectively (Supplementary figure 5a). This was also examined independently within certain gene classes to better reflect a true increase in maturation of the whole repertoire rather than a potential shift in specific subsets driving the increase. For this analysis a significant increase in mean SHM from t1 to t2 was observed in each of the four major gene families represented in this data set: Vh3‐30 (*P* < 0.0001), Vh1‐46 (*P* = 0.0342), Vh3‐9 (*P* = 0.0018) and Vh4‐31 (*P* = 0.0028; Supplementary figure 5c). A similar increase was detected in light‐chain genes Vl1‐47 (*P* = 0.0004) and Vl2‐11 (*P* = 0.0042) in SHM from t1 to t2 (Supplementary figure 5b).

Comparison between disease outcome with both timepoints showed no significant difference between SHM in the S group in comparison with the M group (Figure 3c; S: 3.350 ± 0. 028, M: 3.378 ± 0.028; *P* = 0.5813), whereas an analysis of the mean SHM between t1 and t2 in the S (S–t1: 2.948 ± 0.9640, t2: 3.974 ± 0.9640, *P* < 0.0001) and M (M–t1: 2.537 ± 1.714, t2: 4.380 ± 1.714 *P* < 0.0001) groups reported a significant increase (Figure 3d, e).

We compared SHM levels across the five MBC clusters identified in transcriptomic analysis and found higher SHM in actBC2 when compared with MBC1, MBC2 and actBC1 with *P*‐values of 0.0294, 0.0201 and 0.0036, respectively (Supplementary figure 1e).

Analysis of the clone size indicated an increase in polyclonal population over time, from 91.31% (410/449) at t1 to 93.57% (349/373) at t2 in RBD‐specific MBCs, where about 95% of them were singlets and the rest of the BCRs were part of a clone (*n* = 2 or *n* > 2) at both timepoints (Figure 3f). Twenty‐six clones persisted across all seven patients from t1 to t2 and a significant increase was observed in the SHM of the clones retained over time in both the S (*P* = 0.0025, Figure 3g) and M groups (*P* = 0.0098, Figure 3h).

No statistical difference was observed in CDR3 amino acid length of heavy and light chain from t1 to t2, which was maintained at 15 residues in heavy chain (*P* = 0.8559; Supplementary figure 6a), 9 residues in kappa chain (*P* = 0.2576; Supplementary figure 6b) and 10 residues in lambda chain (*P* = 0.0573; Supplementary figure 6c). Some of the major interactions of antibody–antigen complex are formed with hydrophobic and aromatic residues in the CDR3 region. Thus, we looked at the physicochemical properties (gravy and aromaticity) of CDR3H amino acids across t1 and t2. There was no significant difference in these physicochemical properties from t1 and t2 (gravy *P* = 0.8368, aromaticity *P* = 0.1067; Supplementary figure 6d, e). However, we observed a significant decrease in aromaticity of the clones over time (*P* = 0.0313; Figure 3i) because of changes in CDR3H amino acid residues from aromatic (tyrosine or phenylalanine) residues to polar residues (serine) in the S group at t2. When compared overall, there was no significant change in the gravy index in clones over time (*P* = 0.838; Supplementary figure 6f).

## DISCUSSION

In this longitudinal study of the SARS‐CoV‐2–specific MBCs, several key differences in the single‐cell transcriptomic profile and BCR evolution were observed between three patients with severe COVID‐19, and four with mild/moderate disease up to 4 months after the infection. In these few donors, one notable observation was that the MBCs in the S group displayed increased activation, proliferation and longevity when compared with the M group. These novel findings should be validated in larger cohorts as they may be relevant for understanding long‐term B‐cell induced protection. In addition, both S and M groups displayed an increase in the levels of BCR maturation over time. Together, these results suggest that the long‐term properties of the SARS‐CoV‐2–specific MBCs may vary depending on initial disease severity.

We identified MBC subsets across the groups, which included two activated MBC profiles, *CD80* ^hi^ *TNFAIP3* ^hi^ (actBC1) and *CD11c* ^hi^ *CD95* ^hi^ (actBC2), that have not been previously reported in SARS‐CoV‐2 infection. Interestingly, we observed a sustained decrease in actBC1 in the M group compared with the S and healthy groups. The actBC1 cluster, and the MBCs from the S group in general, maintained a higher expression of genes associated with the TNF‐α signaling via the NF‐κB pathway, suggesting that the B cells in severely ill patients may potentially show better longevity of their SARS‐CoV‐2–specific B cells than the patients with mild‐to‐moderate disease. An activated MBC phenotype has been previously observed in severely infected patients.^12^ These genes in the TNF‐α and NF‐κB pathway are known to be increased in severe COVID‐19 and are a common therapeutic target to reduce the “cytokine storm” in severely infected patients.^31^, ^32^ Increased expression of genes associated with the TNF‐α and NF‐κB pathway in MBCs from the S group is concordant with what has previously been reported in memory CD8 T cells between severe and mild disease.^8^ One major difference though is that the CD8^+^ T cells also had an upregulation of exhaustion markers in the S group. This is in contrast to the MBCs in this study, where the additional gene markers, such as *CD83* and *CD80*, suggest advantageous functions. The frequent observation of T cell lymphopenia, but infrequent B cell lymphopenia, in severe COVID‐19 might account for these observed differences in exhaustion markers between memory T cells and MBCs.

This study revealed a higher expression of *CD83* in the S group. Increased expression of *CD83* has been associated with MBC longevity in mice in adoptive transfer experiments,^33^ but *CD83* overexpression or knockout are both associated with a reduced capacity to proliferate and secrete Ig upon immunization.^34^, ^35^ *CD83* expression has been also shown to be important for anti‐influenza antibody production in the serum, and this may relate to the higher antibody titer that has often been reported in people with more severe disease.^36^

A subset similar to the actBC2 subset has been described in a range of infections, including in SARS‐CoV‐2,^23^ influenza^25^ and other viral infections,^24^ characterized mainly by gene expression of *TBX21* (T‐bet), *FAS* (CD95) and *ITGAX* (CD11c) and reported as being similar to an effector B cell phenotype prone to differentiating into antibody‐secreting cells.^25^, ^37^, ^38^ Our data did not detect *TBX21* expression, perhaps as a result of a technical dropout; however, the features of actBC2 show a similar profile. Higher expression of CD11c has been previously associated with MBCs found in the elderly population in humans and mice, termed age/autoimmune‐associated B cells; however, our actBC2 population differed from age/autoimmune‐associated B cells in the expression of other genes such as *FAS* (CD95) and were not enriched in SARS‐CoV‐2–specific MBCs based on age.^27^, ^39^, ^40^

Our study also revealed a higher expression in the S group of *CD80*. CD80 is associated with providing potent T‐cell help required for antigen presentation and its activation and proliferation for the generation and maturation of GC‐dependent MBCs.^41^, ^42^ A recent study noted that upon vaccination of a group of recovered patients that had predominantly mild infection, no increase in SHM was observed and suggested that these cells may not be re‐entering and proliferating in the germinal centers.^43^ This observation fits with the lower expression of *CD80* seen in our study in the mildly and moderately infected participants.^44^ It would be interesting to determine whether MBCs from patients with more severe illness are more likely to undergo further SHM upon reinfection or vaccination as a result of increased *CD80* expression.

The observation of a general increase in SHM over time in this study is consistent with other recent studies showing the maintenance of germinal centers and antigen stimulation for the ongoing maturation of MBCs up to several months after infection, proving immunity after an infection and after vaccination.^11^, ^43^, ^45^, ^46^ SHM has been reported to be associated with an increase in neutralizing potency; however, this study observed no differences in SHM in the S group in comparison with the M group and similar results have been reported previously.^47^

Further, analysis of the SHM levels of the different clusters indicated that the two activated clusters generally had higher SHM levels than the other clusters. This fits with previous studies showing that these types of activated cells have higher levels of SHM.^25^ A dominant use of IgG MBCs with limited frequencies of IgA MBCs was also reported in this study and was observed at similar frequencies in other studies.^48^, ^49^ It will be important to understand whether the limited frequency of IgA MBCs in the blood will impact on protection from SARS‐CoV‐2 reinfection given that IgA has an important role in protection at the mucosal sites.^50^, ^51^

A key limitation of this study was the small cohort size and number of cells; however, several previous and recent studies have reported similar results in large cohorts.^11^, ^43^ In addition, this study only examined the immune response in the blood and not at the local site of infection. There is currently no consensus on how well immune cells in the blood represent tissue‐resident immune cells, with varying reports of limited through to strong correlations reported.^52^ Given that a recent study reported that there is a substantial frequency of MBCs retained in the lung and lymph nodes up to 6 months after infection,^50^ it would be interesting to understand whether the skewed B cell phenotype we observed in the blood of patients with severe disease is similarly observed at the localized sites, as these are likely the sites that will first respond upon re‐exposure. The spike^+^ B‐cell frequencies reported in this study were lower than the frequency reported for RBD^+^ B cells (Table 1). This was because of a suboptimal concentration (0.25 μg mL^−1^) used in the staining process; however, subsequent studies have reported that this sensitivity is improved with a concentration of 1 μg mL^−1^ of spike tetramer.^14^ In summary, in this study we observed that RBD‐specific MBCs showed varied transcriptional signatures associated with acute COVID‐19 severity that may influence the longevity of the memory responses.

## METHODS

### Data reporting

#### Study design, setting and participants

The COSIN (Collection of COVID‐19 Outbreak Samples in NSW) study is an ongoing prospective cohort study evaluating the natural history of SARS‐CoV‐2 infection among adults and children in New South Wales, Australia. Children and adults diagnosed with SARS‐CoV‐2 infection confirmed by the nucleic acid amplification test were eligible for enrolment, irrespective of disease severity. Participants were enrolled through seven health care services (which provided both inpatient and community‐based care) and their affiliated microbiology laboratories in New South Wales between March 6, 2020 and September 17, 2020. During this time, SARS‐CoV‐2 strains containing the spike protein 614D variant were dominant in Sydney. Follow‐up visits were scheduled at 1 month (visit window: 1–3 months) and 4 months (visit window: 4–6 months) following symptom onset or date of diagnosis (whichever occurred first). At each follow‐up visit, clinical data and blood samples were collected. Disease severity was classified according to the National Institutes of Health stratification (www.covid19treatmentguidelines.nih.gov). The following treatments were provided to the indicated patients while being treated as inpatients: 250002—telmisartan 80 mg mane, amlodipine 5 mg mane, sitagliptin 100 mg mane, allopurinol 1 tablet every 2 days, paracetamol 1 g QID PRN; 289036—perindopril, amlodipine, clotrimazole, prednisone, benzylpenicillin–ceftriaxone–doxycycline and rosuvastatin; 247004—ibuprofen, paracetamol and nebulized saline. Comorbidities were observed in some patients: 250002—hypertension, diabetes and obesity; 289036—chronic lung disease (asthma) and smoking; 250011—obesity; 250017—hypertension and smoking. UC blood samples were collected in 2016 from Australian Red Cross Lifeblood.

### Ethics statement

The protocol was approved by the Human Research Ethics Committees of the Northern Sydney Local Health District and the University of New South Wales, NSW, Australia (ETH00520) and was conducted according to the Declaration of Helsinki and International Conference on Harmonization Good Clinical Practice guidelines and local regulatory requirements. Written informed consent was obtained from all participants before study procedures.

### RBD and spike protein production

The SARS‐CoV‐2 spike RBD (residues 319–541), with an N‐terminal human Ig kappa leader sequence and C‐terminal Avi‐ and His‐tags, was cloned into pCEP4 (Applied Biosystems, Tullamarine, VIC, Australia). Expi293‐Freestyle cells (Applied Biosystems, Tullamarine, VIC, Australia) were cultured at 37°C and 8% CO_2_ in a growth medium containing Expi293 Expression Medium (Applied Biosystems, Tullamarine, VIC, Australia). The plasmid was transiently transfected into Expi293‐Freestyle cells as follows: 1.5 × 10^8^ total cells (50 mL transfection) were mixed with 50 μg of plasmid, 160 μL of ExpiFectamine and 6 mL of Opti‐MEM‐I and left overnight at 37°C in a shaking incubator. The following day, 300 μL of ExpiFectamine Enhancer 1 and 3 mL of ExpiFectamine Enhancer 2 were added to the cells before they were left in culture for a further 48 h. After a total of 72 h in culture, the cell culture was collected and centrifuged for 20 min at 4000*g* at 4°C. Cellular debris was clarified by passing the supernatant two times through a 0.22μm filter. The His‐tagged protein was then affinity purified from the cell supernatant using a HisTrap HP Column (GE Healthcare, Rydalmere, NSW, Australia) and eluted with imidazole (Sigma‐Aldrich, Macquarie Park, NSW, Australia). The purified protein was then buffer exchanged and concentrated in sterile Dulbecco's phosphate‐buffered saline by centrifuging at 4000*g* for 30 min at 4°C in a 10 000 MWCO Vivaspin centrifugal concentrator (Sartorius, Dandenong, VIC, Australia) and stored at −80°C. The recombinant RBD was biotinylated using the AviTag as described by the manufacturer (GeneCopoeia, Gymea, NSW, Australia).

### Isolation of spike and RBD‐specific memory B cells

The tetramerization method was performed as previously described.^14^, ^53^ In brief, biotinylated RBD was incubated with streptavidin–phycoerythrin (Molecular Probes/Thermo Fisher Scientific, Scoresby, VIC, Australia) in a molar ratio of 4:1. The streptavidin dye was added stepwise in one‐tenth volume increments to the biotinylated protein, for a total of 10 times with a 10‐min incubation at 4°C, in a rotating bioreactor, protected from light. Cryopreserved peripheral blood mononuclear cells were thawed rapidly in a 37°C water bath and washed with prewarmed Roswell Park Memorial Institute media (RPMI) supplemented with 2 mm l‐glutamine, 50 IU mL^−1^ penicillin, 50 μg mL^−1^ streptomycin and 10% heat‐inactivated fetal calf serum (Sigma Aldrich, Macquarie Park, NSW, Australia). The cells were resuspended in Dulbecco's phosphate‐buffered saline and counted. All subsequent incubations were performed protected from light. A maximum of 1 × 10^7^ cells were stained with Fixable Viability Stain 700 (FVS700) (1:1000 dilution, BD Bioscience, North Ryde, NSW, Australia) and incubated at 4°C for 20 min, to differentiate the live cells from dead. Cells were washed two times with fluorescence‐activated cell sorting wash buffer (Dulbecco's phosphate‐buffered saline + 0.1% bovine serum albumin), followed by incubation with 5 μL human Fc block per 2 × 10^6^ cells at room temperature for 10 min (BD Biosciences, North Ryde, NSW, Australia), to block nonspecific antibody binding. SARS‐CoV‐2–specific B cells were identified by staining with 1 μg mL^−1^ of RBD tetramer and 0.25 μg mL^−1^ of spike tetramer at 4°C for 30 min. All consecutive steps were performed either at 4°C or on ice and washed two times. The cocktail for staining contained 50 μL stain brilliant buffer and the titrated combination of antibodies: 5 μL each of CD21 BV421, IgD BV510, CD10 BV605, CD19 BV711 and CD20 APC‐H7, 10 μL of IgG BV786, 2 μL each of CD27 PE‐CF594 and CD38 PE‐Cy7, 2.5 μL HLA‐DR BB515 and 0.5 μL CD3 BB700. All the reagents were from BD Biosciences, North Ryde, NSW, Australia. The cells were incubated with the staining cocktail at 4°C for 30 min. They were washed and resuspended in fluorescence‐activated cell sorting wash buffer. A BD FACSAria III sorter was used to phenotype and single cells were sorted (index sorted) into a 96‐well PCR plate containing 2 μL of cold buffer. The buffer was made with lysis buffer containing 0.95 μL of 0.2% Triton X‐100 solution in nuclease‐free water and 0.05 μL recombinant RNase inhibitor (Scientifix, Clayton, VIC, Australia) along with 0.5 μL of 10 mMm deoxynucleotide triphosphate mix (Promega, Alexandria, VIC, Australia), and 0.5 μL of 5 μMm vir70 primer (5′‐AAGCAGTGGTATCAACGCAGAGTACT30VN‐3′, Sigma Aldrich, Macquarie Park, NSW, Australia). The plates were stored in an –80°C freezer. The index data analysis was performed using FlowJo version 10.7.1 (TreeStar).

### Sequencing of RBD‐specific memory B cells

The samples were then RT‐PCR amplified with the Smart‐seq2 approach and sequenced with the Illumina 2 × 150 PE Nextera XT Library Preparation Kit as previously described.^54^, ^55^

### Single‐cell RNA sequencing data analysis

Paired‐end reads from Smart‐seq2 were aligned with STAR (version 2.7.1a) using the GRCh38 human reference genome and transcripts per million were calculated by RSEM (version 1.2.28) using the rsem‐calculate‐expression command on HPC clusters. An in‐house script was used for initial data cleaning and quality control. Genes expressed in none of the cells were removed. Cells with less than 400 expressed genes and more than 30% expressed mitochondrial genes were removed from the matrix. Gene expression matrix was normalized, and batch corrected using Seurat integration implemented in R. Seurat (v4.0.1)^56^ was used to load gene matrix (996 × 17800) comprising IgD^+^ and IgD^−^ SARS‐CoV‐2–specific MBCs for the identification of clusters using the *FindNeighbors()* and *FindClusters()* functions with a resolution of 0.5. DGE analysis between groups (clusters, UC *versus* S group, UC *versus* M group, S *versus* M group) of only IgD^−^ RBD‐specific MBCs was performed using *FindAllMarkers()* with Benjamini–Hochberg–adjusted *P*‐value < 0.05 and log_2_ fold change > 0.1. A Model‐based Analysis of Single‐cell Transcriptomics (MAST) was used for testing the DGE between the groups implemented in Seurat.

### Gene set enrichment analysis

GSEA^57^ was performed using in‐house scripts that use Fast Gene Set Enrichment Analysis version 1.14.0 in R to identify pathways related to genes. The permutations were set to 1000. The databases used to identify pathways in molecular signatures reference platform were Gene Ontology (Biological Processes, Cellular Components, Molecular Function) and Hallmark. Gene signature pathways with *P*‐value < 0.05 and normalized enrichment score > 0 have been significantly upregulated.

### BCR reconstruction

The sequenced samples were used to reconstruct full‐length BCR from VDJpuzzle2.0^54^ by aligning reads with the GRCh38 reference genome. V(d)J genes and framework regions/complementarity‐determining regions were classified according to Igblastn alignments with heavy‐ and light‐chain database present in the IMGT database as part of the VDJPuzzle algorithm. Isotypes were determined according to the VDJPuzzle algorithm by aligning the constant region with the germline constant region sequences from the IMGT database.^58^ In case of IgD^+^ B cells their isotype was annotated as “IgDM” and only highly expressed contigs were considered for the analysis. Change‐O command from Immcantation was used to determine mutations from germline along with clones. Gene usage analysis and physicochemical properties of CDR3H amino acids were calculated using the *countGenes*() and *aminoacidProperties*() commands in alakazam packages version 1.0.2 in R version 3.6.^59^ Phylogenetic tree was reconstructed using full‐length nucleotide sequences of BCR in Clustal Omega for multiple sequence alignment and maximum parsimony tree was reconstructed (https://www.ebi.ac.uk/Tools/msa/clustalo/).^60^ Itolv6 was used to visualize the phylogenetic tree.^61^

## AUTHOR CONTRIBUTIONS

**Money Gupta:** Conceptualization; investigation; methodology; writing – original draft. **Harikrishnan Balachandran:** Methodology. **Raymond Hall Yip Louie:** Investigation; methodology; writing – original draft. **Hui Li:** Methodology; resources. **David Agapiou:** Methodology. **Elizabeth Keoshkerian:** Methodology. **Daniel Christ:** Funding acquisition; writing – review and editing. **William Rawlinson:** Resources. **Michael M Mina:** Funding acquisition; writing – review and editing. **Jeffrey J Post:** Resources; writing – review and editing. **Bernard Hudson:** Resources. **Nicky Gilroy:** Resources. **Pamela Konecny:** Writing – review and editing. **Adam W Bartlett:** Writing – review and editing. **Sarah C Sasson:** Writing – review and editing. **Golo Ahlenstiel:** Resources. **Dominic Dwyer:** Writing – review and editing. **Andrew Lloyd:** Conceptualization; funding acquisition; writing – review and editing. **Marianne Martinello:** Funding acquisition; resources. **Fabio Luciani:** Conceptualization; investigation; methodology; supervision; writing – original draft. **Rowena Bull:** Conceptualization; funding acquisition; investigation; supervision; writing – original draft.

## CONFLICT OF INTEREST

The authors declare no conflicts of interest.

## INCLUSION AND DIVERSITY

We worked to ensure gender balance in the recruitment of human participants. We worked to ensure that the study questionnaires were prepared in an inclusive way. One or more of the authors of this paper self‐identifies/self‐identify as an underrepresented ethnic minority in science. One or more of the authors of this paper self‐identifies/self‐identify as a member of the LGBTQ^+^ community.

## Data Availability

Data are available upon request.
