# Supplementary material for: High activation levels maintained in receptor‐binding domain–specific memory B cells in people with severe coronavirus disease 2019
Source: Immunol Cell Biol. 2022 Dec 1:10.1111/imcb.12607. Online ahead of print. doi: 10.1111/imcb.12607 (PMC9878167; doi:10.1111/imcb.12607)
Supplement: Supplementary file 2 [file IMCB-9999-0-s001.docx]

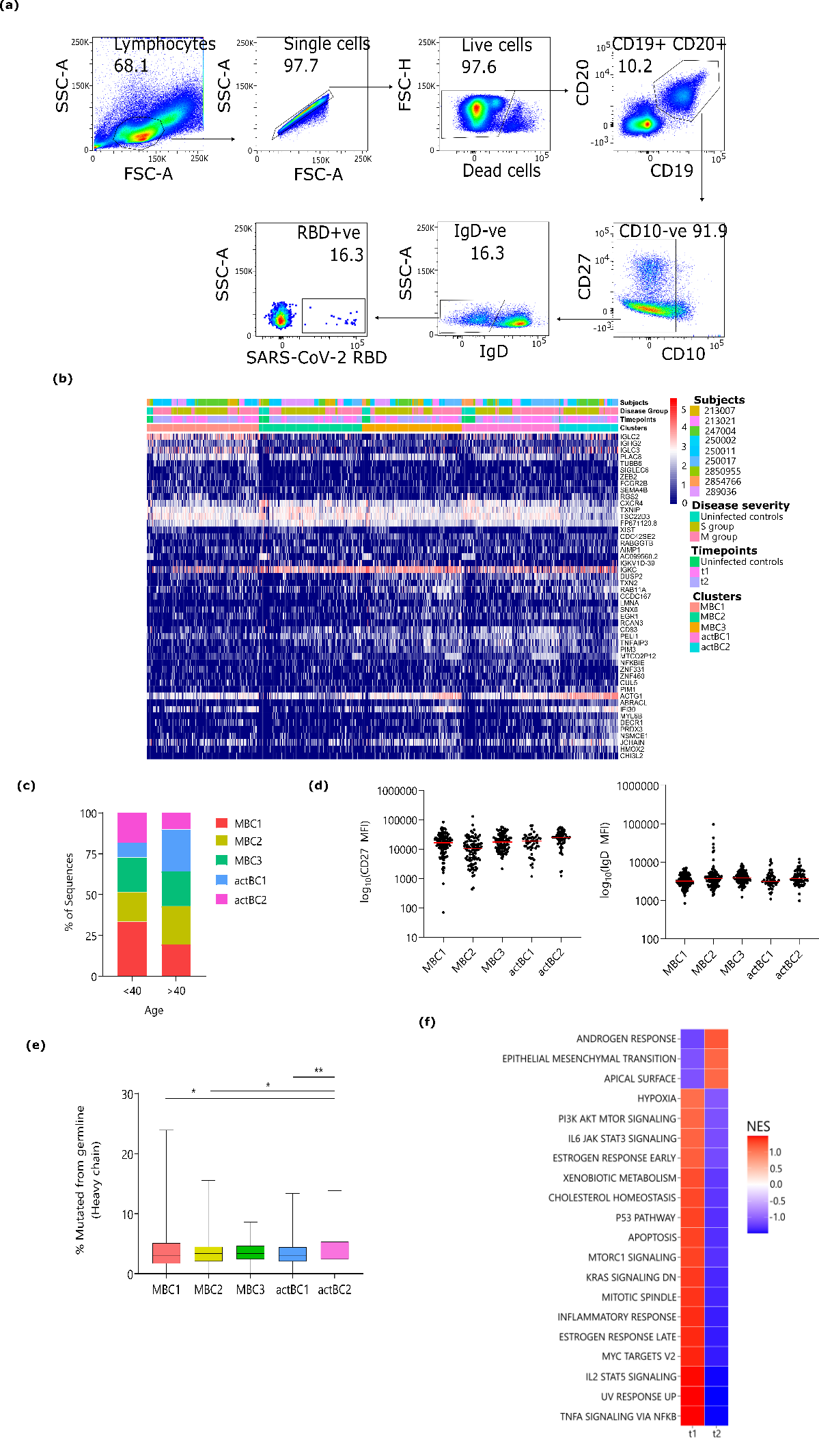


**Supplementary figure 1**. **Characterization of memory B cells across clusters and RBD-specific memory B cells** **(a)** B cell sorting strategy is shown as previously published from our group ^14^. **(b)** Top10 differentially expressed genes across five MBC clusters (MBC1, MBC2, MBC3, actBC1 and actBC2) with metadata showing MBCs across uninfected controls, UC (2850955 and 2854766) and SARS-CoV-2 infected (severe, S group 289036, 247004, 250002; mild/moderate, M group 250011, 213007, 250017 and 213021). **(c)** Proportion of MBC clusters based on subjects with ages less than 40 (UC 2850955; Severe 247004; moderate 213007) and above 40 years (UC 2854766; Severe 289036, 250002; mild 250011, 250017 and 213021). **(d)** Protein mean fluorescent intensity (MFI) of CD27 and IgD showing expression across five clusters (MBC1, MBC2, MBC3, actBC1 and actBC2). **(e)** Percentage mutated from germline in heavy chain across distinct memory B cell clusters. **(f)** Gene Set Enrichment Analysis showing enrichment of pathways in M group across t1 and t2. Statistical differences across SHM in memory B cell clusters were calculated using the two-tailed unpaired *t*-test (Mann-Whitney *U*-test), with adjusted *P*-values **P* < 0.05, ***P* < 0.01.


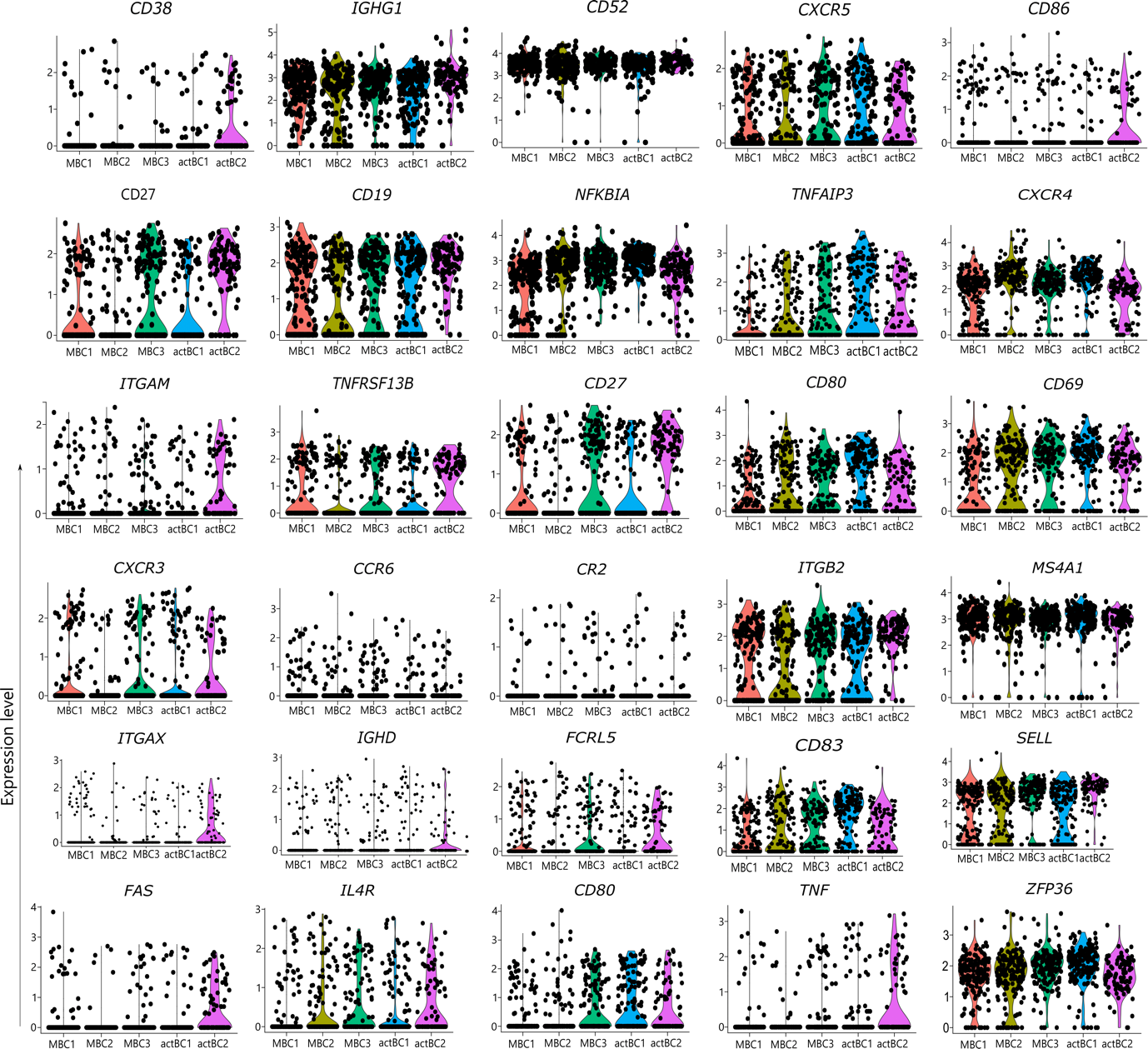


**Supplementary figure 2**. **Gene expression across clusters in RBD-specific memory B cells.** Violin plots across five clusters of MBCs (MBC1, MBC2. MBC3, actBC1, actBC2) showing variability in the expression of activation and housekeeping genes.


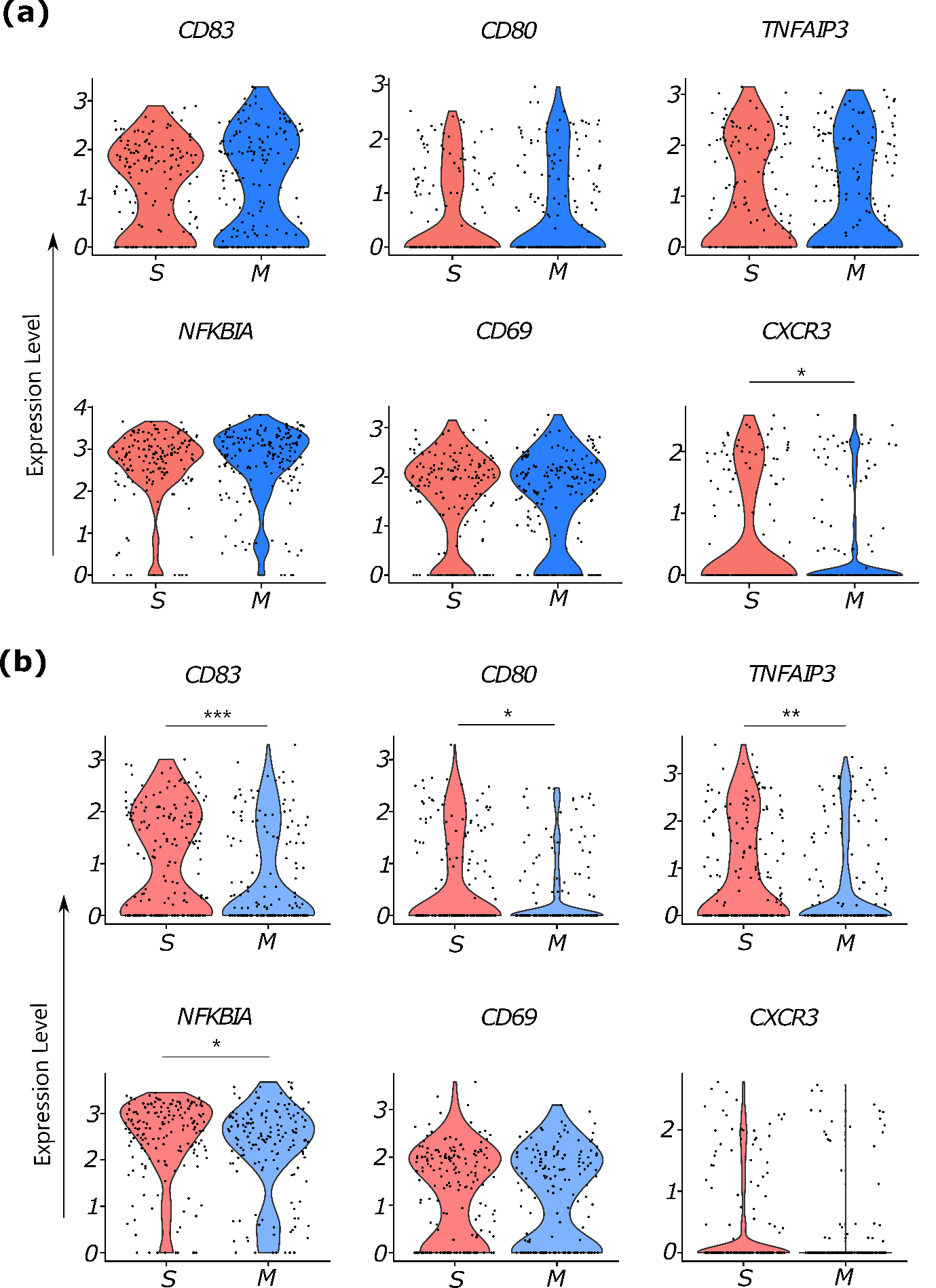


**Supplementary figure 3. Expression level of activation genes across S and M group at t1 and t2 in RBD-specific memory B cells.** **(a)** Violin plots showing variation in gene expression level across S and M group at t1 and **(b)** t2 timepoints. Statistical differences across disease severity were calculated using the unpaired two-tailed Wilcoxon rank sum test with *P*-values depicted as **P* < 0.05, ***P* < 0.01, ****P* < 0.001 and *****P* < 0.0001.


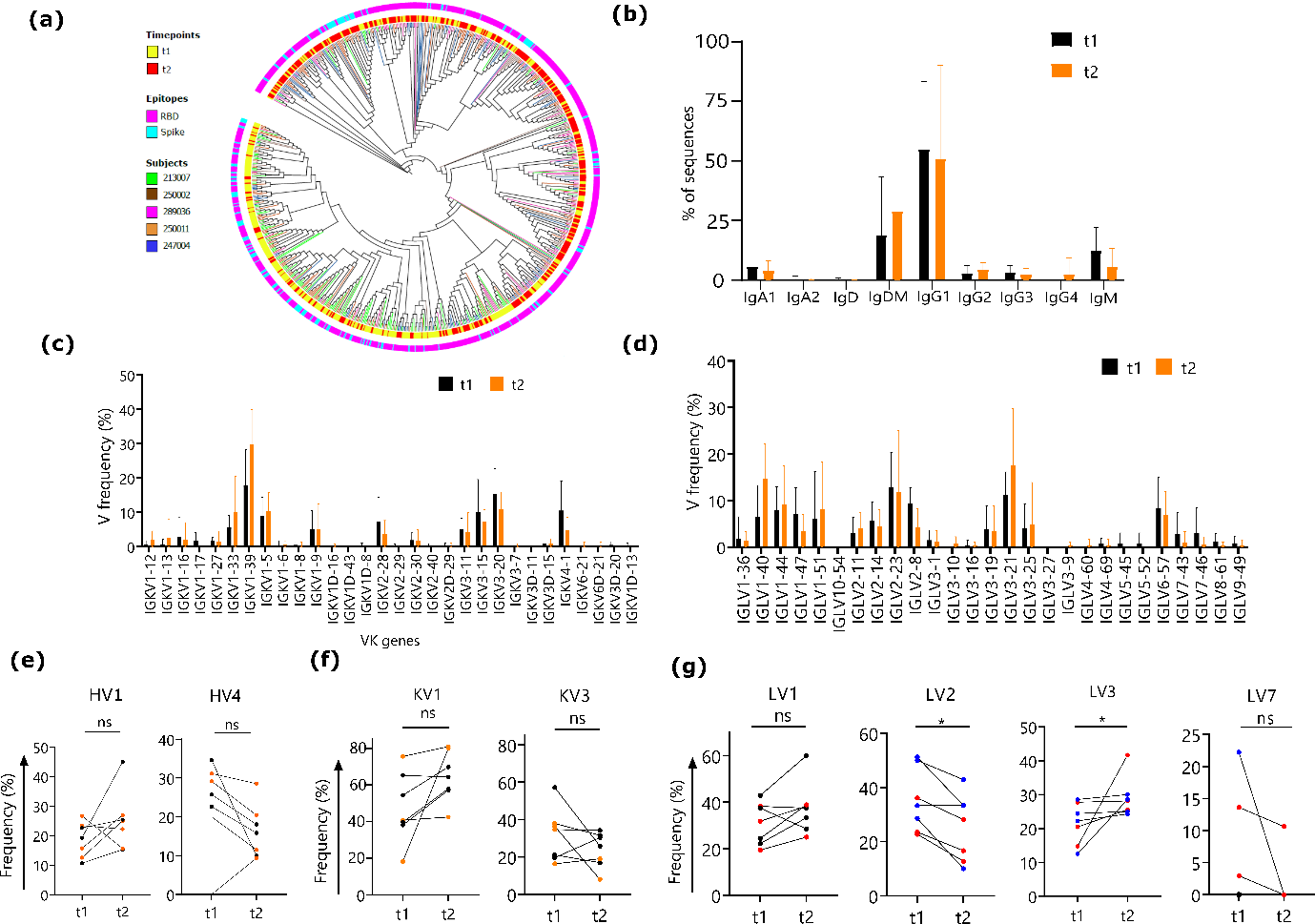


**Supplementary figure 4**. **Characteristics of RBD-specific memory B cell repertoire. (a)** Phylogenetic tree of SARS-CoV-2 specific memory B cells repertoire across infected groups (severe, S 289036, 247004, 250002; mild/moderate, M 250011, 213007, 250017 and 213021) generated using Clustal Omega. Inner strip shows distribution of BCRs (Heavy chain) across two timepoints t1(yellow) and t2(red). Outer strip shows epitope (RBD, pink; Spike, cyan) based distribution of BCRs. **(b)** Bar graph showing percentage of sequences with different isotypes (IgA1, IgA2, IgD, IgDM, IgG1, IgG2, IgG3, IgG4 and IgM) at t1 (black) and t2 (orange). Bar graphs showing RBD-specific B cell receptor with light chain V gene usage in **(c)** kappa and **(d)** lambda chain gene frequency at timepoint t1 (black) and t2 (orange) with mean (+/-SEM). **(e)** Change in percentage frequency of switched, IgD^-^ sequences with HV1 and HV4 **(f)** kappa Chain: KV1 and KV3 and **(g)** lambda chain: LV1, LV2, LV3 and LV7 from t1 to t2 in both Severe, S (black) and mild/moderate, M groups (orange). Statistical differences across disease severity were calculated using the two-tailed unpaired *t*-test (Mann-Whitney *U*-test), adjusted *P*-value as **P* < 0.05, ns = non-significant. The red horizontal line depicts the median at that stage of disease.


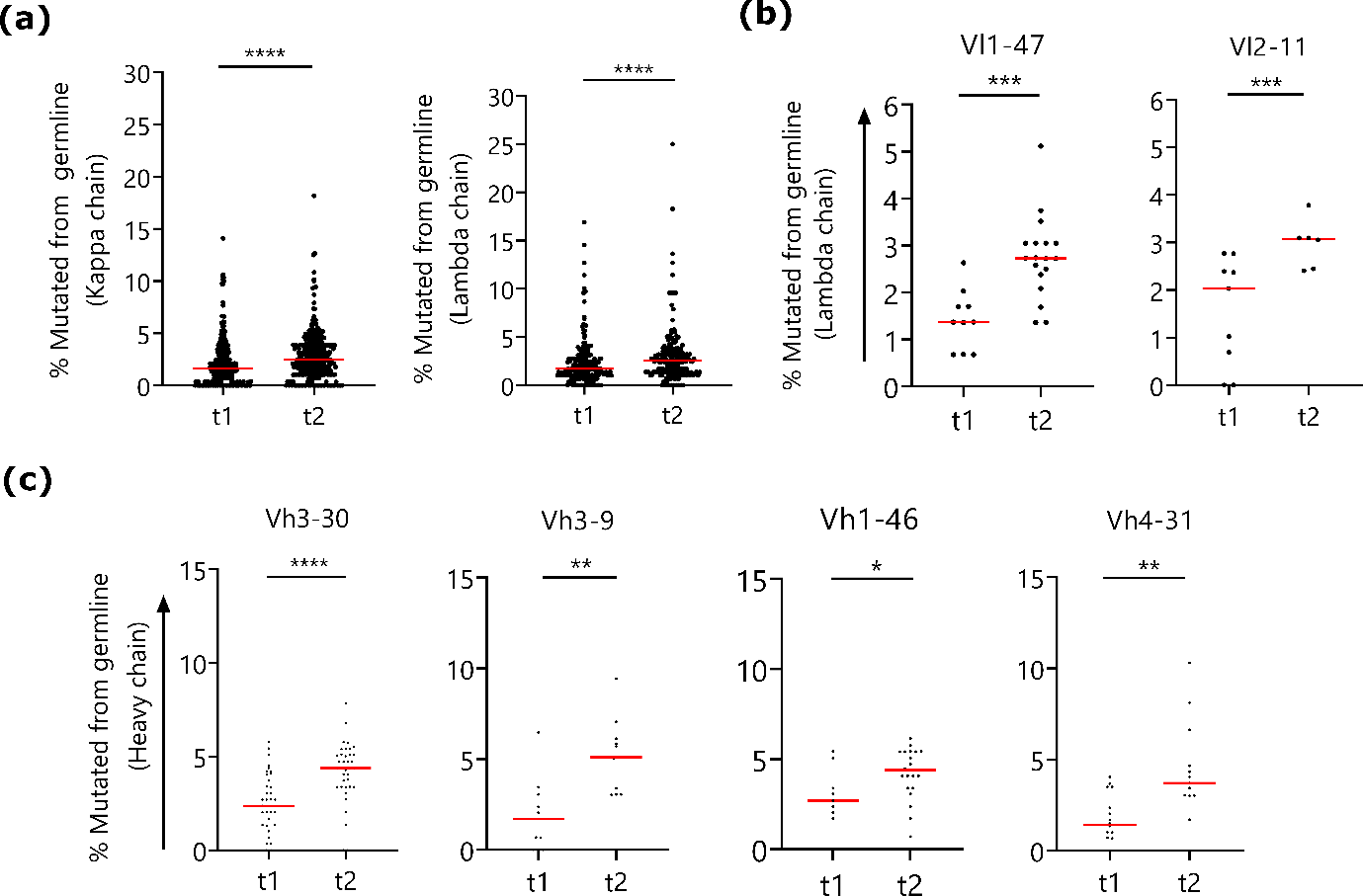


**Supplementary figure 5. Maturation of light chain and gene specific changes in SHM.** **(a)** Percentage change in mutation from germline in Kappa and Lambda chain across t1 and t2. **(b)** Percentage change in mutation from germline in Lambda specific genes Vl1-47 and Vl2-11 and **(c)** and heavy chain genes Vh3-30, Vh1-46, Vh3-9 and Vh4-31.


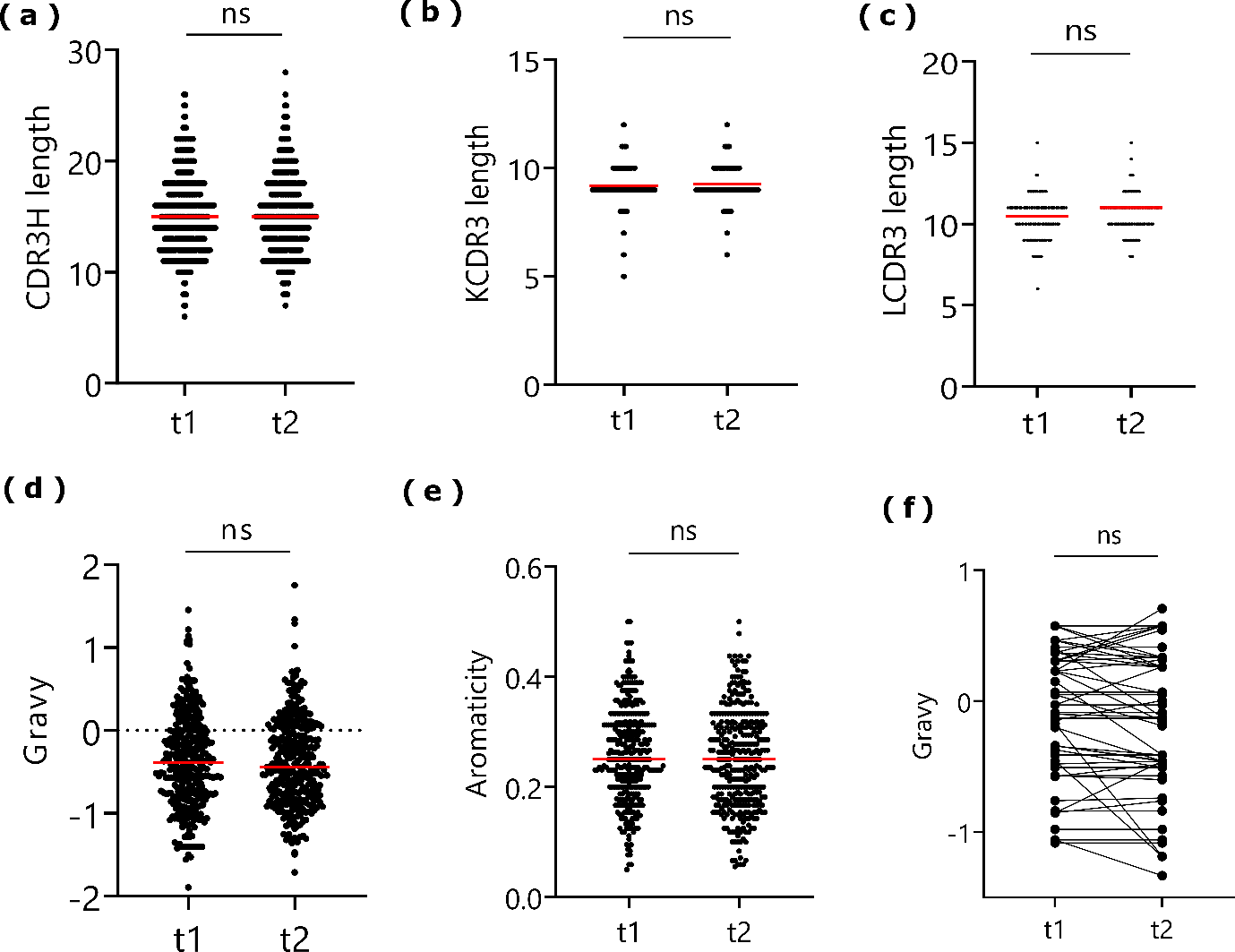


**Supplementary figure 6. Change in CDR3H length and physicochemical properties of RBD-specific Memory B cells.** CDR3 length distribution and physicochemical properties of RBD-specific B cell repertoire. **(a)** CDR3H **(b)** CDR3K and **(c)** CDR3L amino acid sequence length distribution across five subjects across t1 and t2. Physicochemical property of CDR3H amino acid sequence showing change in **(d)** Gravy index and **(e)** aromaticity across t1 to t2. **(f)** Change in gravy index of over time clones across from t1 to t2. Statistical significance was determined using the unpaired two-tailed Mann-Whitney *U*-test. ns = non-significant. The non-parametric paired *t*-test (Wilcoxon) was performed for statistical significance analysis for testing change in gravy index in paired over time clones.
